# Optimal MoCA cutoffs for detecting biologically-defined patients with MCI and early dementia

**DOI:** 10.1007/s10072-022-06422-z

**Published:** 2022-09-28

**Authors:** Ciro Rosario Ilardi, Alina Menichelli, Marco Michelutti, Tatiana Cattaruzza, Paolo Manganotti

**Affiliations:** 1 Department of Psychology, University of Campania “Luigi Vanvitelli”, Viale Ellittico 31, 81100 Caserta, Italy; 2 Neuropsychology Service, Rehabilitation Unit, Department of Medicine, Surgery and Health Sciences, Trieste University Hospital-ASUGI, University of Trieste, Trieste, Italy; 3 Clinical Unit of Neurology, Department of Medicine, Surgery and Health Sciences, Trieste University Hospital-ASUGI, University of Trieste, Trieste, Italy

**Keywords:** Montreal Cognitive Assessment, Mild Cognitive Impairment, Dementia, Cutoff, Sensitivity, Specificity

## Abstract

**Objective:**

In this phase II psychometric study on the Montreal cognitive assessment (MoCA), we tested the clinicometric properties of Italian norms for patients with mild cognitive impairment (PwMCI) and early dementia (PwD) and provided optimal cutoffs for diagnostic purposes.

**Methods:**

Retrospective data collection was performed for consecutive patients with clinically and biologically defined MCI and early dementia. Forty-five patients (24 PwMCI and 21 PwD) and 25 healthy controls were included. Raw MoCA scores were adjusted according to the conventional 1-point correction (Nasreddine) and Italian norms (Conti, Santangelo, Aiello). The diagnostic properties of the original cutoff (< 26) and normative cutoffs, namely, the upper limits (uLs) of equivalent scores (ES) 1, 2, and 3, were evaluated. ROC curve analysis was performed to obtain optimal cutoffs.

**Results:**

The original cutoff demonstrated high sensitivity (0.93 [95% CI 0.84–0.98]) but low specificity (0.44 [0.32–0.56]) in discriminating between patients and controls. Nominal normative cutoffs (ES0 uLs) showed excellent specificity (SP range = 0.96–1.00 [0.88–1.00]) but poor sensitivity (SE range = 0.09–0.24 [0.04–0.36]). The optimal cutoff for Nasreddine’s method was 23.50 (SE = 0.82 [0.71–0.90]; SP = 0.72 [0.60–0.82]). Optimal cutoffs were 20.97, 22.85, and 22.29 (SE range = 0.69–0.73 [0.57–0.83], SP range = 0.88–0.92 [0.77–0.97]) for Conti’s, Santangelo’s, and Aiello’s methods, respectively.

**Conclusion:**

Using the 1-point correction, combined with a cutoff of 23.50, might be useful in ambulatory settings with a large turnout. Our optimal cutoffs can offset the poor sensitivity of Italian cutoffs.

**Supplementary Information:**

The online version contains supplementary material available at 10.1007/s10072-022-06422-z.

## Introduction

Early detection of cognitive impairment in the elderly has never been more relevant in neurological practice, even more so for the newly devised disease-modifying treatments for Alzheimer’s disease (AD), such as the recently FDA-approved Aducanumab [1]. Since AD-modifying therapies might be effective only at stages preceding full-blown dementia, cognitive screening tools should be improved to help clinicians in discriminating between a physiological age-related cognitive decline and prodromal signs imputable to mild cognitive impairment (MCI).

Among the available cognitive screening batteries, the Mini-mental state examination (MMSE [2]) is regarded internationally as a gold standard for assessing global cognitive functioning in moderate/advanced stages of dementia. However, the MMSE weighs heavily on linguistic capabilities, does not sufficiently explore the executive and visuo-spatial/-constructive domains, and may produce false negatives in case of subtle/mild cognitive defects. To deal with such limitations, Nasreddine et al. developed the Montreal cognitive assessment (MoCA) to be coupled with MMSE during neuropsychological evaluation [3]. This is a 30-point screening battery that covers a wide range of cognitive domains, promoting a more in-depth and demanding assessment than MMSE, seemingly more sensitive in detecting patients with MCI and/or early-stage AD [3].

The originally suggested cutoff of 26 provided the best balance between sensitivity and specificity (0.90–1.00 and 0.87, respectively) in distinguishing MCI and AD patients from healthy controls. Nevertheless, several studies have reported an increased false positive rate by using this cutoff value, particularly in older and lower-educated participants [4]. In this regard, the 1-point correction for participants with ≤ 12 years of education has been deemed inadequate for adjusting the nowadays known influence exerted by age and formal schooling [4], as evidenced by the different correction norms available [5–8]. Moreover, some authors have suggested that the inclusion criteria applied in the original investigation for healthy participants (i.e., no cognitive complaints, scores within the normal range at neuropsychological assessment, no abnormalities detected during the neurological examination and CT scan in a subsample of 51 participants) have resulted in a “hyper-normal” control group, with clear repercussions on the cutoff setup [6, 9].

## A European perspective: the Italian experience

The Italian experience involving the MoCA is rather controversial, as is true for the vast majority of psychometric studies in the field of clinical neuropsychology. After release of the Italian-translated version of the MoCA [10], two clinicometric studies [11, 12] attempting to explore the diagnostic properties of the battery and three normative studies [9, 13, 14] defining the ranges of normality in non-clinical populations were published.

### Clinicometric studies

In the Italian clinicometric studies [11, 12], receiver operating characteristic (ROC) curve analysis was used to assess the diagnostic accuracy of the MoCA. Pirrotta et al. [11] tested 287 participants living in the Sicily region (Southern Italy) and split the whole sample into two groups based on the MMSE score (i.e., experimental group, *n* = 154, MMSE score < 26; control group, *n* = 133, MMSE score > 26). The authors indicated a cutoff of 15.5 as the best threshold value for distinguishing between the two groups (sensitivity = 0.83, specificity = 0.97). This study, however, presents some criticisms. First, an arbitrary MMSE cutoff was used to perform the splitting; however, updated adjusting norms and a weighted cutoff (i.e., 23.8) were already available on cross-sectional data collected in a representative normative sample [15]. Second, the experimental and control groups were not equivalent in terms of years of age and formal schooling. Indeed, participants within the experimental group were older and less educated than those included in the control group (*M* age = 76.8 vs. 63.1; *M* education = 6.0 vs. 10.4). Finally, although the concomitant effects of higher age and lower education had already been observed in the Italian population [16], analyses were conducted on raw scores.

Bosco et al. [12] tested the diagnostic accuracy of the MoCA by comparing AD patients with age-and-education-matched controls from Southern Italy. A cutoff ≤ 14 was found to effectively discriminate between patients and healthy controls (sensitivity = 0.87, specificity = 0.87–0.92). In addition, the authors calculated the best MoCA cutoff value (MoCA score ≤ 17, sensitivity = 0.86, specificity = 0.63) for differentiating older adults with suspected cognitive impairment (i.e., adjusted MMSE score ≤ 23.8) from those with spared cognitive functioning (i.e., adjusted MMSE score > 23.8). Although the authors applied the propensity score matching procedure [12, 17] in order to minimize the effects of sociodemographic variables on the diagnostic accuracy estimation of the MoCA, their study included only participants with poor education. Furthermore, using a state variable based on the MMSE cutoff may not be an acceptable solution when exploring the diagnostic properties of a cognitive tool since the outcome of a screening assessment may be not sufficient for properly characterizing a cognitive profile [18].

### Normative studies

As argued before, normative data taking into account the effects of potentially confounding demographic variables are needed to correctly interpret the MoCA score, i.e., to compare patients’ scores apart from demographic differences. Since 2015, three independent Italian contributions [9, 13, 14] providing regression-based norms [19–21] have been published.

Conti et al. [9] extracted norms from a sample consisting of 225 healthy older adults (*M* age = 70.1 years, *SD* = 5.7, range = 60–80; *M* education = 9.9 years, SD = 4.6, range = 5–23) residing in the Emilia Romagna region (Northern Italy). The mean raw MoCA score was 23.28 (SD = 3.22).

In the same year, Santangelo et al. [13] provided normative data from a larger sample (*N* = 415) that spanned age groups ranging from 21 to 95 years (*M* age = 56.82 years, SD = 18.8), leading to an expected increase in the average years of education (*M* education = 11.13 years, SD = 4.76, range = 1–21). Participants were healthy volunteers mainly recruited in Naples (Southern Italy) and in some other districts of Central and Northern Italy. In this study, the mean raw MoCA score was 21.98 (SD = 4.22).

More recently, updated MoCA norms have been provided by Aiello et al. [14]. Their sample involved a total of 579 healthy participants (*M* age = 63.44 years, SD = 15.04, range = 21–96; *M* education = 11.27 years, SD = 4.6, range = 1–25) from the Lombardia region (Northern Italy). The authors reported an average raw MoCA score of 24.17 (SD = 3.93).

The above studies present discrepancies. For instance, in Santangelo’s study, the mean raw MoCA score was slightly lower than reported by Conti et al., likely due to the inclusion of 48 elderly over 80 s (*M* = 17.08, SD = 4.90) inflating the variance and lowering the average score. Moreover, Santangelo’s normative sample included only 158 participants with ages between 60 and 79 years [13]. Still, Aiello et al.’s participants achieved a raw MoCA score higher than that reported in the other two normative studies; in this respect, the greater number of units under 60 years of age (*n* = 335/579) may have played a role [14]. These methodological issues, in combination with inter-regional socio-demographic and cultural heterogeneity, are likely reflected in the differences observed in correction factors and normative cutoffs.

### Aims

In the clinical neuropsychology literature, most of the psychometric studies have historically been focused on providing normative data, i.e., on investigating how raw scores are distributed in the normal population, whether raw scores are affected by confounding variables, and what the normality values are (e.g., normative cutoff). These contributions (phase I psychometric studies), however, are necessary but not sufficient; indeed, they should be followed by accurate clinicometric studies (phase II psychometric studies) in which the test’s discriminative capability and diagnostic properties of optimal cutoffs (e.g., sensitivity, specificity, predictive values, accuracy) are estimated. Clearly, much depends on the objectives of the neuropsychological evaluation. For instance, phase II psychometric studies are needed when the aim is to assess the presence of a clinical condition (e.g., MCI or dementia) but less so when the purpose is to delineate the cognitive profile in patients with an established pathology (e.g., traumatic brain injury) or to monitor cognitive functioning over time. From a purely diagnostic perspective, phase II psychometric studies are helpful in determining if normative cutoffs can be declined in clinical practice. To this end, diagnostic properties of normative and optimal cutoffs can be compared: the former is the test score above which, typically, there should be approximately 95% of the normal population (with 95% confidence interval) [21], while the latter is the value ensuring the best balance between sensitivity and specificity in discriminating patients from controls [22].

In Italy, the two available clinicometric studies on the MoCA are not the “physiological” evolution of the three normative studies. This is also the reason why no consensus exists on which cutoff to use in outpatient services. Accordingly, in order to offset this gap, the aim of the present study is to test the clinicometric properties of the Italian MoCA’s normative data, using Nasreddine’s method as a gold standard international reference. We also provide optimal cutoffs for each adjusting method under examination. To the best of our knowledge, this is the first study on the matter.

## Materials and methods

### Participants and procedure

Retrospective data collection was performed for consecutive patients with suspected MCI previously referred to the Memory Centre of the Trieste University Hospital (Neurological Unit, Azienda Sanitaria Universitaria Integrata Giuliano Isontina, ASUGI) for diagnostic and treatment purposes, from 2018 to 2021. Patients fulfilled the following inclusion criteria: age < 85 years, at least 5 years of formal schooling, clinical dementia rating (CDR [23]) score ≥ 0.5, and clinically diagnosed MCI [24] or early dementia, according to functional impairment. The etiological diagnosis was supported by National Institute on Aging and Alzheimer’s Association guidelines (NIA-AA-2011 [25]) for AD, Rascovsky’s criteria [26] for behavioral variant frontotemporal dementia (bvFTD), IWG-2 criteria [27] for mixed AD (i.e., in case AD pathology with concurrent signs of cerebrovascular disease), DLB Consortium criteria [28] for Dementia with Lewy Bodies (DLB), and International Society for Vascular Behavioral and Cognitive Disorders (VASCOG) Criteria [29] for vascular cognitive disorder (VCD).

All patients underwent a comprehensive clinical assessment consisting of extensive neuropsychological evaluation (Supplementary Material 1), general physical and neurological examination, laboratory testing (i.e., thyroid, liver and kidney function, B12, folate, homocysteine, electrolytes, and blood cells count), neuroimaging scans (CT or MRI), EEG, SPECT/CT or PET/CT, and lumbar puncture to test cerebrospinal fluid AD biomarkers (Aβ42, t-Tau, and p-Tau; Aβ40 and Aβ42/40 ratio when available). It is important to underline that MoCA was not part of the neuropsychological composite battery employed to support MCI or dementia diagnosis. Exclusion criteria were moderate/advanced stage of dementia disease, severe vascular encephalopathy, acquired brain lesions, other neurodegenerative disease (e.g., primary progressive aphasia), psychiatric disorders (e.g., major depression), history of alcohol abuse and/or psychotropic drug therapies, and learning disabilities.

Out of a total of 144 eligible patients, 99 were excluded as they were only given the MMSE (i.e., no data on the MoCA were available). Therefore, 45 patients were included in the study. Of these, 24 patients met clinical diagnosis of MCI (PwMCI; amnestic MCI-multiple domain, *n* = 21; nonamnestic MCI-single domain with selective executive impairment, *n* = 2; nonamnestic MCI-multiple domain, *n* = 1); the remaining 21 patients were diagnosed with early-stage dementia (PwD; AD, *n* = 6; mixed AD, *n* = 10; FTD, *n* = 5). In line with the abovementioned criteria for etiological diagnosis, among MCI patients, 15 were diagnosed with MCI due to AD (MCI-AD; five of them with mixed vascular pathology), 6 were diagnosed with mild VCD, 2 with MCI-DLB, and 1 patient with MCI-FTD.

In addition, a sample of healthy older adults (HCs) was enrolled as control group. Thirty volunteers were recruited in different districts within Friuli-Venezia Giulia, Veneto, and Trentino-Alto Adige regions. Inclusion criteria were age < 85 years, years of formal education ≥ 5, CDR score = 0, MMSE score ≥ 28, and no cognitive complaints. Exclusion criteria were previous or current neurocognitive, psychiatric, or psychopathological disorders, and ongoing treatments with psychotropic medications interfering with cognition. Candidates with pharmacologically compensated chronic medical illnesses (e.g., hypertension, type II diabetes, cardiovascular diseases) were not excluded to reduce the risk of a “hyper-normality” bias. Of the 30 candidates, 5 were excluded according to the above criteria.

Both patients and HCs completed the Italian version of the MoCA. In short, the MoCA consists of twelve subtests exploring spatial–temporal orientation, short- and verbal long-term memory, visuospatial and visuoconstructional abilities, language skills, sustained attention, and different executive domains, i.e., set-shifting, working memory, verbal fluency, and abstraction capabilities. The administration takes about 10 min.

### Statistical analysis

Descriptive statistics, expressed as frequency (sex) or mean and standard deviation (age, education, raw MoCA score), were stratified according to subgroups. Between-group comparisons were performed by two-way chi-square test (*χ*^2^) for nominal variables and univariate analysis of variance (ANOVA) for continuous variables prior determination of univariate normality according to skewness and kurtosis indexes. Any post hoc analysis was conducted using Bonferroni’s method.

Raw MoCA scores were separately adjusted according to four independent correction methods, namely, the traditional 1-point correction by Nasreddine et al. and the correction factors for age and education reported in the Italian normative studies by Conti et al., Santangelo et al., and Aiello et al. For the sake of clarity, we computed four independent adjusted MoCA scores for each participant. Then, a general descriptive cutoff analysis was conducted by comparing the adjusted MoCA scores to the respective reference cutoffs. The conventional threshold value of 26 was used as a touchstone for 1-point adjusted MoCA scores; conversely, age-and-education adjusted MoCA scores were compared to the related Italian normative cutoffs, namely, the upper limits (uLs) of the equivalent scores (ES) 1, 2, and 3. ES are derived from a 5-point ordinal scale, from 0 (adjusted score < 5th centile) to 4 (adjusted score ≥ 50th centile). Particularly, ES1, 2, and 3 are obtained by dividing the adjusted distribution between ES0 and ES4 into three equal parts. ES uLs correspond to the non-parametric outer tolerance limits (e.g., in the case of ES0, the outer tolerance limit on the 5th centile [20, 21]).

In the general cutoff analysis, the test results — or predicted conditions (positive vs. negative) — were contrasted with the true conditions (present vs. absent disease) for all participants. The resulting outcomes were true positive (TP), true negative (TN), false positive (FP), and false negative (FN). Based on the count produced for each outcome, the following parameters — reflecting the test’s diagnostic capability — were estimated: sensitivity (SE) = $\frac{TP}{(TP+FN)}$ (true positive rate, i.e., the proportion of patients for which the predicted condition was positive), specificity (SP) = $\frac{TN}{(TN+FP)}$ (true negative rate, i.e., the proportion of HCs for which the predicted condition was negative), positive predictive value (PPV) = $\frac{TP}{(TP+FP)}$ (i.e., the proportion of participants with a predicted positive condition for which the true condition was positive), negative predictive value (NPV) = $\frac{TN}{(TN+FN)}$ (i.e., the proportion of participants with a predicted negative condition for which the true condition was negative), false positive rate (FPR) = $\frac{FP}{(FP+TN)}$ (fall-out, i.e., the proportion of HCs for which the predicted condition was positive), false negative rate (FNR) = $\frac{FN}{(FN+TP)}$ (miss rate, i.e., the proportion of patients for which the predicted condition was negative), and accuracy (ACC) = $\frac{(TP+TN)}{(TP+TN+FP+FN)}$ (i.e., the total proportion of correctly classified participants). As concerns sensitivity and specificity, 95% confidence intervals were calculated in accordance with the continuity-corrected score method [30, 31].

A nonparametric ROC curve analysis was performed to (i) assess the diagnostic accuracy (or discriminatory power) of the four MoCA adjusting methods, i.e., to evaluate the extent to which the four MoCA’s adjusted scores could discriminate between patients and HCs, and (ii) to determine the optimal cutoff value (also known as optimal decision threshold) for each of the four methods across all the possible cutoff points based on a simultaneous assessment of sensitivity and specificity [32].

According to agreed conventions, an area under the ROC curve (AUC) between 0.50 and 0.70 is considered weakly discriminative, values within the range 0.70–0.80 are considered acceptable, whereas an AUC higher than 0.80 is deemed excellent [33]. The results of a priori power analysis [34] suggested that, at a nominal alpha level of 0.05, power set to 0.80, minimum expected AUC of 0.70, and an allocation ratio equal to 1, the required total sample size was 48 (24 patients vs. 24 HCs).

For each ROC-estimated cutoff, the same parameters used for characterizing the normative cutoffs were calculated. In addition, the Youden index (YI, sensitivity + specificity – 1) was used to identify the optimal cutoff values. The YI-based method defines an optimal cutoff as the value that maximizes the difference between sensitivity and FPR, i.e., the vertical distance between the 45° line and the point on the ROC curve [22, 35]. The higher the YI, the better the cutoff. Normative and ROC-estimated cutoffs were descriptively compared.

Statistical analyses were performed using IBM SPSS Statistics for Windows, version 26.0 (IBM, Armonk, NY), Stata Statistical Software, release 15 (StataCorp LLC, College Station, TX), and easyROC (R language), version 1.3.1. The nominal alpha level was set to 0.05.

## Results

### Descriptive statistics

As shown in Table 1, patient (PwMCI and PwD) and control groups were matched for sex (*χ*^2^_1_ = 0.56, *p* = 0.45), age (patients, *M* = 71.98, SD = 5.79, range = 57–83; HCs, *M* = 69.32, SD = 9.12, range = 58–86; *F*_1, 68_ = 2.222, *p* = 0.14), and years of formal schooling (patients, *M* = 11.93, SD = 4.27, range = 5–18; HCs, *M* = 10.80, SD = 4.55, range = 5–18; *F*_1, 68_ = 1.081, *p* = 0.30). Furthermore, no sociodemographic differences emerged when comparing HCs, PwMCI, and PwD (sex, *χ*^2^_2_ = 1.717, *p* = 0.42; age, *F*_2, 67_ = 1.105, *p* = 0.34; education, *F*_2, 67_ = 0.548, *p* = 0.58). As for the MoCA score, unsurprisingly, the control group obtained a mean raw score higher than the patient group (controls, *M* = 24.36, SD = 3.29, range = 18–30; patients, *M* = 20.09, SD = 3.73, range = 9–26; *F*_1, 68_ = 22.568, *p* < 0.001, *η*^*2*^ = 0.25, 1–*β* = 1.00); furthermore, HCs outperformed both PwMCI and PwD (*F*_2, 67_ = 13.607, *p* < 0.001, *η*^*2*^ = 0.29, 1–*β* = 1.00; HCs vs. PwMCI, *M* diff. = 3.318, *p* = 0.005; HCs vs. PwD, *M* diff. = 5.36, *p* < 0.001). Conversely, no difference was found between PwMCI and PwD (*M* diff. = 2.042, *p* = 0.17). These findings suggest overlapping characteristics of the target populations — both in terms of sociodemographic variables and global cognitive functioning — and warrant our choice to perform an in-depth analysis on the MoCA’s diagnostic capability regardless of the patient subgroups. In other words, we merged the two patient subgroups and assessed the clinicometric performance of normative and optimal cutoffs in distinguishing HCs from PwMCI and PwD.

**Table 1:** Descriptive statistics

Sample characteristics	Overall patients	MCI (CDR 0.5)	Early dementia (CDR ≥ 1)	Healthy controls (CDR 0)	Comparisons
Sex (f/m)^a^	21/24	13/11	8/13	14/11	HCs = Pts; HCs = PwMCI = PwD
Age (years)^b^	71.98 (5.79)	71.83 (6.09)	72.14 (5.58)	69.32 (9.12)	HCs = Pts; HCs = PwMCI = PwD
Education (years)^b^	11.93 (4.27)	12.04 (4.07)	11.81 (4.58)	10.80 (4.55)	HCs = Pts; HCs = PwMCI = PwD
MoCA (raw)^b^	20.09 (3.76)	21.04 (2.58)	19.00 (4.60)	24.36 (3.29)	HCs > Pts; HCs > PwMCI = PwD

*MoCA* Montreal cognitive assessment, *MCI* mild cognitive impairment, *CDR* clinical dementia rating scale, *Pts* total patients, *PwMCI* patients with mild cognitive impairment, *PwD* patients with dementia, *HCs* healthy controls

Mean (SD)

^a^Pearson’s *χ*^2^ test

^b^ANOVA

### Cutoff analysis on the normative values

The results of the general cutoff analysis involving normative values are reported in Table 2. The original cutoff of 26 demonstrated high sensitivity (SE = 0.93) but low specificity (SP = 0.44), resulting in FPR inflation (FPR = 0.56). However, Nasreddine’s cutoff showed, as a whole, a fair degree of classification capability (ACC = 0.75).

**Table 2:** Results of general analysis on normative cutoffs

Normative cutoffs	Patients (*n* = 45)		Healthy controls (*n* = 25)		PPV	NPV	FPR	FNR	ACC
	T + /T–	Sensitivity (95% CI)	T + /T–	Specificity (95% CI)					
Nasreddine et al. (2005)									
< 26	42/3	0.93 (0.84–0.98)	14/11	0.44 (0.32–0.56)	0.75	0.79	0.56	0.07	0.75
Conti et al. (2015)									
ES0 uL ≤ 17.36	9/36	0.20 (0.12–0.32)	0/25	1.00 (0.93–1.00)	1.00	0.41	0.00	0.80	0.48
ES1 uL < 19.50	23/22	0.51 (0.39–0.63)	2/23	0.92 (0.82–0.97)	0.92	0.51	0.08	0.49	0.66
ES2 uL < 21.56	31/14	0.69 (0.57–0.79)	5/20	0.80 (0.68–0.88)	0.86	0.59	0.20	0.31	0.73
ES3 uL < 23.36	40/5	0.89 (0.79–0.95)	13/12	0.48 (0.36–0.60)	0.75	0.71	0.52	0.11	0.74
Santangelo et al. (2015)									
ES0 uL ≤ 15.50	4/41	0.09 (0.04–0.19)	0/25	1.00 (0.93–1.00)	1.00	0.38	0.00	0.91	0.41
ES1 uL < 18.28	10/35	0.22 (0.13–0.34)	0/25	1.00 (0.93–1.00)	1.00	0.42	0.00	0.78	0.50
ES2 uL < 20.25	17/28	0.38 (0.27–0.50)	1/24	0.96 (0.88–0.99)	0.94	0.46	0.04	0.62	0.58
ES3 uL < 22.23	25/20	0.56 (0.43–0.67)	1/24	0.96 (0.88–0.99)	0.96	0.54	0.04	0.44	0.70
Aiello et al. (2022)									
ES0 uL ≤ 18.58	11/34	0.24 (0.15–0.36)	1/24	0.96 (0.88–0.99)	0.92	0.41	0.04	0.75	0.50
ES1 uL < 20.69	22/23	0.49 (0.37–0.61)	1/24	0.96 (0.88–0.99)	0.96	0.51	0.04	0.51	0.66
ES2 uL < 22.56	33/12	0.73 (0.61–0.83)	7/18	0.72 (0.60–0.82)	0.82	0.60	0.28	0.27	0.73
ES3 uL < 24.52	39/6	0.87 (0.76–0.93)	10/15	0.60 (0.48–0.71)	0.80	0.71	0.40	0.13	0.77

*ES* equivalent score, *uL* upper limit, *T* + positive test result, *T–* negative test result, *PPV* positive predictive value, *NPV* negative predictive value, *FPR* false positive rate, *FNR* false negative rate, *ACC* accuracy

The ES0 uL by Conti et al. allowed to correctly identify all the HCs (SP = 1.00); nevertheless, it showed a very low sensitivity (SE = 0.20, FNR = 0.80). Among the cutoff points proposed by Conti et al., the ES2 uL appeared to be the most effective (SE = 0.69, SP = 0.80, ACC = 0.73).

Similar to Conti’s normative data, the ES0 uL by Santangelo et al. demonstrated perfect specificity but extremely poor sensitivity (SE = 0.09, FNR = 0.91), with the cutoff point that was able to correctly classify only 4 out of 45 patients. Among the Santangelo’s cutoff values, the best compromise was the ES3 uL, although its diagnostic sensitivity was still far too low (SE = 0.56, SP = 0.96, ACC = 0.70). In return, the ES3 uL showed an excellent PPV. Indeed, by using this cutoff point as a reference, a positive condition was predicted for 26 participants, of which 25 were patients with neurocognitive impairment (PPV = 0.96).

Finally, the ES0 uL by Aiello et al. showed high specificity (SP = 0.96) but low sensitivity (SE = 0.24), resulting in an FNR of 0.75. Among the cutoff points reported by Aiello et al., the ES2 uL guaranteed good sensitivity and specificity, downsizing false outcomes, and increasing classification accuracy (SE = 0.73, SP = 0.72, FPR = 0.28, FNR = 0.27, ACC = 0.73).

### ROC curve analysis and optimal cutoffs

A ROC curve analysis was performed to quantify, for each of the four adjusting methods, the MoCA’s capability in discriminating patients (merged group of PwMCI and PwD) from HCs, and to determine the optimal cutoffs. Irrespective of the method used, the MoCA proved to be highly discriminative (all AUCs > 0.80, *p*_s_ < 0.001, see Table 3), with no difference detected between the four AUCs according to the equality test (*χ*^2^_3_ = 1.56, *p* = 0.67).

**Table 3:** Area under the receiver operating characteristic (ROC) curve for each method

Adjusting method	AUC	SE	*p*-value	95% CI^a^
Nasreddine et al. (2005)	0.81	0.05	< 0.001	0.70–0.90
Conti et al. (2015)	0.83	0.05	< 0.001	0.72–0.91
Santangelo et al. (2015)	0.85	0.05	< 0.001	0.74–0.92
Aiello et al. (2022)	0.84	0.05	< 0.001	0.74–0.92

*AUC* area under curve, *SE* standard error

^a^Binomial exact confidence interval

The ROC-estimated cutoff values are displayed and characterized in Table 4. About Nasreddine’s method, the optimal cutoff was 23.50 (SE = 0.82, SP = 0.72, ACC = 0.78, YI = 0.54). As compared with the original cutoff of 26, this lower cutoff substantially gains in terms of specificity, flattening the FPR and maximizing the diagnostic accuracy.

**Table 4:** Diagnostic properties of ROC-based cutoffs for each adjusting method

ROC-based cutoffs	Patients (*n* = 45)		Healthy controls (*n* = 25)		PPV	NPV	FPR	FNR	ACC	YI
	T + /T–	Sensitivity (95% CI)	T + /T–	Specificity (95% CI)						
Nasreddine et al. (2005)										
≤ 14.50	4/41	0.09 (0.04–0.19)	0/25	1.00 (0.93–1.00)	1.00	0.38	0.00	0.91	0.41	0.09
≤ 18.50	10/35	0.22 (0.13–0.34)	0/25	1.00 (0.93–1.00)	1.00	0.42	0.00	0.78	0.50	0.22
≤ 21.50	25/20	0.56 (0.43–0.67)	4/21	0.84 (0.73–0.91)	0.86	0.51	0.16	0.44	0.66	0.40
**≤ 23.50**	**37/8**	**0.82 (0.71–0.90)**	**7/18**	**0.72 (0.60–0.82)**	**0.84**	**0.69**	**0.28**	**0.18**	**0.78**	**0.54**
≤ 25.50	42/3	0.93 (0.84–0.98)	14/11	0.44 (0.32–0.56)	0.75	0.79	0.56	0.07	0.75	0.37
≤ 26.50	44/1	0.98 (0.90–1.00)	18/7	0.28 (0.18–0.40)	0.71	0.87	0.72	0.02	0.73	0.26
≤ 27.50	45/0	1.00 (0.93–1.00)	20/5	0.20 (0.12–0.32)	0.69	1.00	0.80	0.00	0.71	0.20
Conti et al. (2015)										
≤ 17.93	11/34	0.24 (0.15–0.36)	0/25	1.00 (0.93–1.00)	1.00	0.42	0.00	0.75	0.51	0.24
≤ 19.14	22/23	0.49 (0.37–0.61)	1/24	0.96 (0.88–0.99)	0.96	0.51	0.04	0.51	0.65	0.45
** ≤ 20.97**	**31/14**	**0.69 (0.57–0.79)**	**3/22**	**0.88 (0.77–0.94)**	**0.91**	**0.61**	**0.12**	**0.31**	**0.75**	**0.57**
≤ 21.55	31/14	0.69 (0.57–0.79)	5/20	0.80 (0.68–0.88)	0.86	0.59	0.20	0.31	0.73	0.49
≤ 22.58	36/9	0.80 (0.68–0.88)	9/16	0.64 (0.52–0.75)	0.80	0.64	0.36	0.20	0.74	0.44
≤ 24.30	42/3	0.93 (0.84–0.98)	15/10	0.40 (0.29–0.52)	0.74	0.77	0.60	0.07	0.74	0.33
≤ 26.90	45/0	1.00 (0.93–1.00)	22/3	0.12 (0.06–0.22)	0.67	1.00	0.88	0.00	0.68	0.12
Santangelo et al. (2015)										
≤ 16.65	6/39	0.13 (0.07–0.24)	0/25	1.00 (0.93–1.00)	1.00	0.39	0.00	0.87	0.44	0.13
≤ 18.56	11/34	0.24 (0.15–0.36)	0/25	1.00 (0.93–1.00)	1.00	0.42	0.00	0.75	0.51	0.24
≤ 21.31	21/24	0.47 (0.35–0.59)	1/24	0.96 (0.88–0.99)	0.95	0.50	0.04	0.53	0.64	0.43
**≤ 22.85**	**32/13**	**0.71 (0.59–0.81)**	**2/23**	**0.92 (0.82–0.97)**	**0.94**	**0.64**	**0.08**	**0.29**	**0.78**	**0.63**
≤ 24.28	36/9	0.80 (0.68–0.88)	9/16	0.64 (0.52–0.75)	0.80	0.64	0.36	0.20	0.74	0.44
≤ 26.42	40/5	0.89 (0.79–0.95)	15/10	0.40 (0.29–0.52)	0.73	0.67	0.60	0.11	0.71	0.29
≤ 27.81	45/0	1.00 (0.93–1.00)	17/8	0.32 (0.22–0.44)	0.73	1.00	0.68	0.00	0.76	0.32
Aiello et al. (2022)										
≤ 18.70	11/34	0.24 (0.15–0.36)	1/24	0.96 (0.88–0.99)	0.92	0.41	0.04	0.75	0.50	0.20
≤ 20.67	22/23	0.49 (0.37–0.61)	1/24	0.96 (0.88–0.99)	0.96	0.51	0.04	0.75	0.51	0.45
** ≤ 22.29**	**33/12**	**0.73 (0.61–0.83)**	**3/22**	**0.88 (0.77–0.94)**	**0.92**	**0.65**	**0.12**	**0.27**	**0.78**	**0.61**
≤ 23.55	37/8	0.82 (0.71–0.90)	9/16	0.64 (0.52–0.75)	0.80	0.67	0.36	0.18	0.75	0.46
≤ 25.17	40/5	0.89 (0.79–0.95)	16/9	0.36 (0.25–0.48)	0.71	0.64	0.64	0.11	0.70	0.25
≤ 26.78	45/0	1.00 (0.93–1.00)	18/7	0.28 (0.18–0.40)	0.71	1.00	0.72	0.00	0.74	0.28
≤ 28.01	45/0	1.00 (0.93–1.00)	23/2	0.08 (0.03–0.18)	0.66	1.00	0.92	0.00	0.67	0.08

*T* + positive test result, *T–* negative test result, *PPV* positive predictive value, *NPV* negative predictive value, *FPR* false positive rate, *FNR* false negative rate, *ACC* accuracy, *YI* Youden index

Optimal cutoffs, and their diagnostic properties, are displayed in bold

As for Conti’s method, the optimal cutoff was 20.97 (SE = 0.69, SP = 0.88, ACC = 0.75, YI = 0.57). If compared with the nominal normative cutoff (i.e., ES0 uL = 17.36), this threshold value — which is almost halfway between uLs of ES1 and 2 — has better sensitivity, thus reducing the FNR; moreover, it ensures a higher specificity and increases accuracy.

About Santangelo’s method, the optimal cutoff was 22.85 (SE = 0.71, SP = 0.92, ACC = 0.78, YI = 0.63). This cutoff is about seven points above the nominal normative cutoff (i.e., ES0 uL = 15.50) and slightly higher than the ES3 uL. As compared to the former, a cutoff point of 22.85 greatly increases sensitivity while maintaining excellent specificity. Accordingly, this cutoff dramatically flattens the FNR and maximizes diagnostic accuracy. As compared to the latter, it does guarantee higher sensitivity and accuracy.

**Fig. 1:**
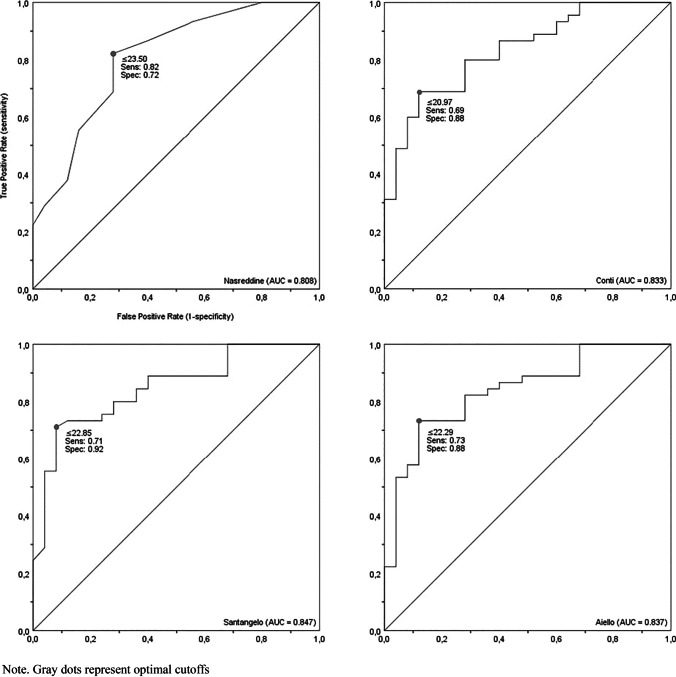
Receiver operating characteristic (ROC) curves for each of the four adjusting method used Note. Gray dots represent optimal cutoffs

Finally, about Aiello’s method, the optimal cutoff was 22.29 (SE = 0.73, SP = 0.88, ACC = 0.78, YI = 0.61). This cutoff approaches the ES2 uL; however, it demonstrates higher specificity, thus reducing the FPR. If compared with the nominal cutoff point (i.e., ES0 uL = 18.58), a higher threshold value of 22.29 significantly increases both test sensitivity, resulting in a lowered FNR, and diagnostic accuracy. ROC curves and the respective optimal cutoffs are shown in Fig. 1.

## Discussion

As outlined in a recent systematic review [36], the custom of assessing psychometric properties of cognitive tests on the normal population, without verifying their diagnostic properties on target conditions, prevails in Italy and beyond. The establishment of this approach likely stems from historic overconfidence in the effectiveness of normative data, allowing to quickly capture normality values for screening purposes. Nevertheless, normative cutoffs are generally unweighted for sensitivity and specificity, and this makes it unclear whether they are truly useful in clinical practice when the actual goal is to identify a specific pathology (such as MCI or dementia).

The MoCA is a short and easy-to-administer screening battery used worldwide for supporting the diagnosis of MCI and early dementia, as well as for detecting cognitive deficits in patients with Parkinson’s disease, stroke, chronic obstructive pulmonary disease, or heart failure [37]. Despite its popularity, in Italy, there is still no consensus about the cutoff(s) to be used, even more so for the mixed psychometric evidence [9, 11–14]. Accordingly, in the present study, we performed the first phase II psychometric study on a sample consisting of PwMCI, PwD, and HCs. First, we adjusted separately each raw MoCA score in compliance with the correction factors provided in the Italian normative studies by Conti et al. [9], Santangelo et al. [13], and Aiello et al. [14]; furthermore, raw scores were also adjusted according to the 1-point basic correction by Nasreddine et al. [3] for a total of four independent adjusted scores for each participant. Then, we compared the diagnostic properties of Italian normative values with sensitivity- and specificity-weighted (optimal) cutoffs computed via ROC analysis, using Nasreddine’s correction method and the original cutoff (< 26/30 points) as international references.

For both normative and optimal cutoffs, we calculated relevant indexes of diagnostic capability, all of which have a significant impact on clinical practice. For instance, heavy emphasis should be placed on the role of predictive values. Given the result of a test, the PPV is the likelihood that a subject with a positive test actually has the condition of interest; conversely, the NPV is the likelihood that a subject with a negative test result is truly free of such a condition. Predictive values are affected by the test’s sensitivity and specificity: the more sensitive the test (lower FNR), the greater the NPV; the more specific the test (lower FPR), the greater the PPV. A high PPV is desirable, by and large, when the costs for performing the test are high or in the case of a slowly worsening disease. A high NPV is instead preferred in the context of serious health conditions or when the disease can be treated or delayed if managed in the early stages [38].

In line with previous studies [4], we found that using a cutoff of 26 as the threshold value for 1-point-adjusted MoCA scores entailed poor specificity (0.44), leading to an inflated FPR (0.56). Conversely, the cutoff yielding the best diagnostic performance for Nasreddine’s method was 23.50, a value close to that recommended in a recent meta-analysis on the diagnostic properties of the MoCA (i.e., 23 [4]). This cutoff minimized false positives (0.28) and guaranteed a good balance between sensitivity (0.82) and specificity (0.72). Therefore, the use of Nasreddine’s method, combined with a cutoff of 23.50, might represent a viable alternative to Italian normative criteria, particularly in ambulatory settings with a large turnout. In such a settings, this quick adjusting method may indeed ensure some gains in terms of time needed to correct raw scores. However, this hypothesis must be treated with caution since demographic effects are only weakly addressed by the classical 1-point correction [7, 39].

As for Italian norms [9, 13, 14], nominal cutoffs (ES0 uL) showed the opposite outcomes to Nasreddine’s cutoff, namely, excellent specificity but very low sensitivity (i.e., 0.09–0.24). This result is due to the statistical approach used in the normative studies, which provides for the assignment of ES0 uLs to the outer tolerance limits on the fifth centile of adjusted distributions, in order to decrease the inferential risk of false positives [21]. Nonetheless, it is important to stress that the choice of a reference cutoff should be guided by the assessment aims. For instance, in experimental designs posing cognitive impairment as an exclusion criterion, lower cutoffs should be preferred as they guarantee higher specificity and hence fewer false positives. On the contrary, in clinical settings, where decreasing the risk of false negatives is imperative (e.g., neuropsychological assessment for diagnostic purposes or clinical trials), a higher cutoff should be preferred to increase diagnostic sensitivity [40].

The ES2 uLs reported by Conti et al. and Aiello et al. demonstrated, on the whole, good diagnostic properties, broadly comparable to those observed for ROC-estimated cutoffs, with the latter providing, however, higher specificity (Conti: 0.80 vs. 0.88, Aiello: 0.72 vs. 0.88). Since optimal cutoffs for Conti’s (≤ 20.97)^1^ and Aiello’s (≤ 22.29) norms were located between ES1 and ES2 uLs, we suggest that future psychometric studies aiming at providing normative values for distinguishing HCs from PwMCI/PwD may raise nominal cutoffs towards innermost regions of the adjusted distributions, e.g., between the 20th and 35th centiles (or, in terms of *z*-deviates, between –1.24 and –0.62 [19, 20]), as long as the sample size is sufficiently large. However, there are some issues to be taken into account.

Typically, normative cutoffs serve to identify potential deficits that are conventionally associated with the score distribution slice where the “worst” 5% of the healthy population is found. This occurs similarly to what happens in statistical tests when the nominal alpha is set to 0.05 for controlling the type I error. By definition, this corresponds to the incorrect rejection of a true null hypothesis. Declining the principle in the clinical-neuropsychological setting, a cut-point set at the 5th centile allows decreasing the risk of false positive, i.e., the risk of mistakenly rejecting the null hypothesis that an individual is free from impairment. This approach clearly increases the test specificity and therefore the FNR. Moving, for instance, the alpha from 0.05 to 0.20, the statistical power increases and the risk of making the type II error is reduced. This error occurs when one fails to reject a false null hypothesis, e.g., classifying a subject with cognitive deficits as healthy. In diagnostic terms, decreasing the risk of committing the type II error means flattening the FNR by increasing the test sensitivity and therefore the number of false positives (i.e., the risk of type I error grows). Putting the matter in practical terms, on the one hand, a higher cutoff could be actually more sensitive in detecting PwMCI/PwD in clinical contexts to which individuals with suspected cognitive impairment refer. On the other hand, using a higher threshold in the context of a general cognitive screening addressed to a random population may lead to excessively inflated FPR.

The case of Santangelo et al. is apparently controversial as not even the ES3 uL (< 22.23) reached an acceptable sensitivity (0.56). The questionable diagnostic properties of these cutoffs might be explained by some intrinsic characteristics of the normative sample including mainly participants residing in Southern Italy [13]. Accordingly, since our study involved only participants from Northern Italy, inter-regional differences may have played a confounding role. In the face of these limitations, the optimal cutoff point for Santangelo’s method (≤ 22.85) showed satisfactory sensitivity (0.71) and specificity (0.92), and the highest PPV (0.94) and YI (0.63) among optimal cutoffs. Therefore, although larger clinicometric investigations are needed to test the goodness of Santangelo et al.’s normative data, we suggest that their correction factors might be confidently applied, in combination with a cutoff of 22.85, by neurologists and neuropsychologists working in Northern Italy.

Because of time constraints and over-working, clinicians may often suffer from a “representativeness heuristic” bias affecting their judgments in selecting the normative data to be deployed in clinical practice. They might rely on specific norms based on the normative sample size, the patient populations of interest, or the newest published paper. Although new versions of neuropsychological tools generally have advantages over prior versions, such as improved psychometric properties or administration procedures [41], inter-regional socio-demographic and cultural differences within the same country may be relevant confounding predictors [14, 42]. The example of Santangelo’s normative data, which are currently used in both clinical and research environments of Northern Italy [43, 44], reflects on the need for a national commitment that supports multicentric studies aimed at collecting large-scale normative data for cognitive tests, taking into account, for instance, differences in cultural background, educational quality, language, communication style, occupational level, economic issues, cognitive reserve, and intellectual functioning [45]. Interestingly, a recent study by Montemurro et al. [46] providing MoCA’s normative data and clinical cutoffs for the Italian population highlighted that both sociodemographic variables and cognitive reserve predicted the variance of the MoCA score. These normative values were not tested in the present investigation since we covered only normative studies correcting raw scores for sex, age, and education and reporting unique correction factors/equivalent scores according to the classical regression-based procedures. Accordingly, future clinicometric studies are needed to further assess the role of cognitive reserve in predicting cognitive performance.

The current study has some limits. The sample size is restricted; however, particularly for ROC analysis, preliminary estimations ensured adequate statistical power with a minimum of 48 participants. A further threat to external validity is that we applied a retrospective nonprobability sampling method, and the consecutive patient subsample could not homogeneously cover all the diagnostic categories of clinical interest. Still, previous psychometric investigations on the MoCA have reported differentiated optimal cutoffs for MCI and dementia [47]. Since we found no difference between PwMCI and PwD on the MoCA score, we provided optimal cutoffs for distinguishing controls from patients independently on the disease stage. Possible explanations for our null-finding are the modulating roles of cognitive reserve [48], socio-cultural attitudes [49], or reliability of caregiver’s ratings on the patient’s functional status [50], which can be affected by a number of factors such as caregiver burden [51] and time spent on assisting the patient [52]. It follows that performance below our optimal cutoffs indicates that the patient can suffer from either MCI or early dementia. Therefore, an in-depth diagnostic investigation is still needed; nevertheless, it would be mandatory given the screening nature of the battery. Finally, we provided predictive values based on prevalence estimates derived from the sample addressed. Therefore, these may diverge from those highlighted within a potential replication study.

## Conclusions

In sum, this study demonstrated that all the Italian available adjusting methods for the MoCA were highly discriminative when comparing an independent sample of HCs and a sample including both PwMCI and PwD. Nevertheless, as expected, nominal normative cutoffs yielded high specificity but at cost of sensitivity, determining an increased rate of false negatives when the goal is to detect the presence of a clinical condition such as MCI or dementia. This can have repercussions on healthcare cost-effectiveness and patients’ management, especially in the current historical period where the early detection of PwMCI is of paramount importance as ideal candidates for future AD-modifying treatments. To improve the diagnostic capabilities of normative data, we suggest implementing adjustments to the well-established equivalent scores method, and to perform nationwide normative studies for controlling biases due to inter-regional differences.

## Supplementary Information

Below is the link to the electronic supplementary material.

Supplementary file1 (DOCX 21 KB)

## Footnotes

1 Note that the lower optimal cutoff for Conti’s method is probably the result of severer correction factors (i.e., they reward less and penalize more), given the normative sample including only elderly people [9].
