# Supplementary material for: Optimal MoCA cutoffs for detecting biologically-defined patients with MCI and early dementia
Source: Neurol Sci. 2022 Sep 28;44(1):159–70. doi: 10.1007/s10072-022-06422-z (PMC9816212; doi:10.1007/s10072-022-06422-z)
Supplement: Supplementary file 1 — Supplementary file1 (DOCX 21 KB) [file 10072_2022_6422_MOESM1_ESM.docx]

**Supplementary Material 1**

List of tests administered during neuropsychological evaluation

| Domains | Test | Reference |
| --- | --- | --- |
| General Cognitive Functioning |  |  |
|  | Mini Mental State Examination | Measso et al. (1993) |
|  | Clock Drawing Test | Caffarra et al. (2011) |
| Short-Term Memory |  |  |
| Verbal | Digit Span Forward | Monaco et al. (2013) |
| Visuospatial | Corsi Block Task Forward | Monaco et al. (2013) |
| Working Memory |  |  |
| Verbal | Digit Span Backward | Monaco et al. (2013) |
| Visuospatial | Corsi Block Task Backward | Monaco et al. (2013) |
| Episodic Memory |  |  |
| Verbal | Semantically Unrelated Word-List (Immediate Recall, Delayed Recall, Recognition, Rate of Forgetting) | Mauri et al. (1997) |
| Visuospatial | Rey-Osterrieth Complex Figure (Delayed Recall) | Caffarra et al. (2022) |
| Attention and Executive Functioning |  |  |
| Selective Attention, Inhibition of Automatic Responses, and Processing Speed | Stroop Color and Word Test (Color Items, Color-Word Items) | Brugnolo et al. (2016) |
| Set-Shifting | Trail Making Test (Part A, Part B, Part B-A) | Giovagnoli et al. (2008) |
|  |  | Siciliano et al. (2019) |
| Abstract Reasoning, Mental flexibility, and Problem Solving | Modified Wisconsin Card Sorting Test | Caffarra et al. (2004) |
| Cognitive Estimation, | Cognitive Estimation Task | Scarpina et al. (2015) |
| Divergent Thinking and Creative Cognition |  |  |
|  | Modified Five-Point Test | Cattelani et al. (2011) |
|  |  | Battista et al. (2021) |
| Social Cognition |  |  |
| Cognitive and Affective Theory of Mind | Story-based Empathy  Task | [Dodich](https://pubmed.ncbi.nlm.nih.gov/?term=Dodich+A&cauthor_id=26072203) et al. (2015) |
| Language |  |  |
| Lexical Retrieval, Production, and Initiation | Phonemic Fluency (FAS) | Costa et al. (2014) |
|  | Semantic Fluency | Costa et al. (2014) |
|  | Oral Naming | Catricalà et al. (2013) |
| Visuospatial Abilities |  |  |
| Visuospatial Functions, Visuomotor Coordination, Constructional Praxis, Planning, and Organizational Skills | Rey-Osterrieth Figure-Copy | Caffarra et al. (2002) |
| Behavioral Disturbances |  |  |
|  | Neuropsychiatric Inventory | Cummings et al. (1994) |
|  | Frontal  Behavioural Inventory | Alberici et al. (2007) |
| Functional Autonomies |  |  |
|  | Basic Activities of Daily Living | Katz et al. (1963) |
|  | Instrumental Activities of Daily Living | Lawton and Brody (1969) |
